# Adapting evidence-based psychological treatments for inpatient care: Commentary on Rudd (2025)

**DOI:** 10.1017/S0033291725101621

**Published:** 2025-08-28

**Authors:** Mariah T. Hawes, Stephanie Marcello, Evan M. Kleiman

**Affiliations:** 1 Rutgers University Behavioral Health Care, Piscataway, New Jersey, USA; 2 Department of Psychology, Rutgers, The State University of New Jersey, Piscataway, NJ, USA

## Abstract

Rudd (2025) submitted a comment on our recent editorial, which highlights the widespread lack of access to evidence-based psychological treatment (EBT) in psychiatric inpatient care and encourages increased efforts to study adaptations of existing EBTs for this setting (Hawes, Marcello, & Kleiman, 2025). In our editorial, we specifically call for investment in inpatient group therapy, as this is the dominant mode of psychological treatment in psychiatric hospitals due to limited staff availability coupled with a lack of reimbursement incentives for individual therapy. Rudd offers a compelling case for the consideration of problem-specific and strategic treatment add-ons adapted from existing EBTs for inpatient care, such as his adaptation of brief cognitive behavioral therapy (CBT) for suicide prevention for inpatient care (BCBT-I). BCBT-I is an abbreviated version of a one-on-one outpatient EBT that produced comparable reductions in posttreatment suicide risk to the full protocol (Diefenbach et al., 2024). We agree that existing EBTs that can serve as efficient add-ons to traditional care, such as BCBT-I, can offer a compelling avenue for improving inpatient care. We view adaptations of EBTs for group therapy and strategic add-on formats as complementary and overlapping strategies that together address the ongoing crisis in inpatient care. In this article, we elaborate on the challenges in adapting EBTs for acute settings and how nontraditional treatment models, like strategic treatment add-ons and open, stand-alone single-session groups, can address these challenges.

Rudd (2025) submitted a comment on our recent editorial, which highlights the widespread lack of access to evidence-based psychological treatment (EBT) in psychiatric inpatient care and encourages increased efforts to study adaptations of existing EBTs for this setting (Hawes, Marcello, & Kleiman, 2025). In our editorial, we specifically call for investment in inpatient group therapy, as this is the dominant mode of psychological treatment in psychiatric hospitals due to limited staff availability coupled with a lack of reimbursement incentives for individual therapy. Rudd offers a compelling case for the consideration of problem-specific and strategic treatment add-ons adapted from existing EBTs for inpatient care, such as his adaptation of brief cognitive behavioral therapy (CBT) for suicide prevention for inpatient care (BCBT-I). BCBT-I is an abbreviated version of a one-on-one outpatient EBT that produced comparable reductions in posttreatment suicide risk to the full protocol (Diefenbach et al., 2024). We agree that existing EBTs that can serve as efficient add-ons to traditional care, such as BCBT-I, can offer a compelling avenue for improving inpatient care. We view adaptations of EBTs for group therapy and strategic add-on formats as complementary and overlapping strategies that together address the ongoing crisis in inpatient care. In this article, we elaborate on the challenges in adapting EBTs for acute settings and how nontraditional treatment models, like strategic treatment add-ons and open, stand-alone single-session groups, can address these challenges.

## Challenges

The prototypical psychological EBT is between 12 and 20 sessions of weekly, outpatient individual therapy, though several outpatient group interventions follow a similar timeline. As highlighted in our editorial and reiterated by Rudd (2025), time demands in inpatient care are considerable. The typical inpatient stay (3–7 days) is significantly shorter than the standard number of EBT sessions, even if sessions are administered on consecutive days, which they are not designed to be. Furthermore, significant time is spent integrating into the unit at admission and discharge planning at the end of the stay. The logistics of unit scheduling are chaotic, limiting the consistency and continuity of treatment. Therapy groups are frequently canceled last-minute due to unexpected staff conflicts, such as when multiple patients require one-on-one observation for safety concerns or when clinicians are pulled into meetings (e.g. with family members or for discharge planning). Individual sessions are typically not formally integrated into the unit schedule, instead occurring whenever staff and patients have availability, which sometimes involves pulling patients out of a group session. Frequent patient ‘pull-outs’ from the group to complete other unit tasks is a long-standing gripe of inpatient group therapists and is exacerbated in units whose leadership does not value group therapy (Yalom, 1983). Moreover, discharge decisions are often made within 24–48 hours, so clinicians embarking on a course of individual therapy have little idea of how many sessions they will be able to fit into the patient’s stay. Together, these factors contribute to a highly unpredictable and limited therapeutic window.

Other factors that contribute to the challenges of adapting EBTs for inpatient settings include patient severity and restrictions (or lack thereof) on the group therapy format. Inpatients, compared to outpatients, are more impaired overall, presenting with more severe mental illness diagnoses (e.g. psychosis, bipolar disorder, personality pathology) and higher comorbidity of psychological and medical conditions (Gobbicchi et al., 2021; Goldman et al., 2020). Due to high patient turnover, groups are forced to be open format, allowing patients to enter at any session in the group series, whereas most outpatient groups are closed format with all patients entering/concluding treatment together. Furthermore, inpatient groups often involve all willing individuals on the unit at a given time (a consequence of unit size and staff availability), which results in a heterogeneous mix of patient needs, unlike outpatient groups, which are typically targeted to specific problems (e.g. depression).

## Recommendations

Given these complexities and limitations, inpatient psychotherapy necessarily looks very different than outpatient psychotherapy. Considering the short length-of-stay and scheduling inconsistencies, inpatient psychotherapy needs to be very *brief* and *flexible.* Furthermore, the heterogeneity and overall severity of patient presentations paradoxically pull for treatment that is both highly *individualized* and *generalized.* No single treatment can meet all of these needs. Thus, we suggest that the best approach to psychological treatment in inpatient care is a multimodal approach that incorporates multiple types of psychotherapies delivered in various formats. This approach has long been applied to other aspects of inpatient care. In addition to the emphasis on psychopharmacological treatment, unit schedules are packed with psychoeducational and life skills groups, peer support groups, art and physical exercise activities, and unit/community meetings (Yalom, 1983). Each activity targets different patient needs, and together they holistically support patients’ wellness. Many of these activities are labeled as ‘therapies’; however, few are based on psychological EBTs, and, as outlined in our editorial, hardly any of these group adaptations have been empirically evaluated (Hawes et al., 2025). There is also precedent from outpatient treatment models, such as the coupling of individual therapy and skills group in dialectical behavioral therapy (Linehan, 2015). There is a clear need to integrate evidence-based psychological models and techniques into the existing multimodal approach to inpatient care.

What could this look like in practice? As we have reiterated, group therapy is the dominant and most practical format for inpatient psychological treatment. In our editorial, we argued that inpatient groups should be *open* (no restrictions on who attends), designed in a *single-session* format (knowledge from prior group sessions is not required to benefit from the current session), and based on *transdiagnostic* treatment models. In contrast to the problem-specific treatment add-ons suggested by Rudd (2025), inpatient groups are more appropriate for addressing generalized patient needs. Groups are an ideal format to teach transdiagnostic conceptual models from EBTs, such as the organization of experience into thoughts, feelings, and behaviors, and understanding of how these interact with each other and the environment from the CBT model.

Strategic treatment add-ons, on the other hand, have the potential to allow for greater individualization of treatment, targeting specific patient problems such as suicide risk, substance use, or eating pathology. One of the strengths of BCBT-I that Rudd (2025) highlights, which could apply to strategic treatment add-ons in general, is that, because it is based on the fundamental CBT model, it can easily be integrated with other CBT-based treatments within the unit. For example, when paired with a group teaching, a common conceptual model and fundamental treatment skills, individual sessions are likely to be much more efficient, focusing on applying the model to the patient’s unique problems and troubleshooting implementation of skills. In this way, inpatient groups and strategic treatment add-ons can be complementary. However, they may also overlap, as Rudd (2025) suggests that strategic treatment add-ons could be delivered in a small group format. Small, specialized groups that are less diagnostically or symptomatically heterogeneous than those typically seen in open group formats allow for greater depth of discussion on a particular topic and more individual patient attention.

A last important consideration for integrating group therapy and strategic treatment add-ons is staff availability and training. Offering problem-specific individual and small group sessions alongside the standard unit-wide group sessions will depend on the availability of staff; yet, this option remains far more feasible than trying to implement EBTs following their typical timeline and scope. As we note in our editorial, therapy groups are often run by staff who have a limited background in EBTs, such as psychiatric nurses (Hawes et al., 2025). At face value, generalized therapy groups appear easier for minimally trained staff to deliver, especially when highly structured and focused on a simple conceptual model with a few predetermined skills. On the other hand, specialized individual and small group interventions likely require clinicians with more extensive training in EBTs who have the ability to tailor the interventions for each patient. For example, BCBT-I involves conducting a narrative analysis of a recent suicide attempt, and then developing an individualized plan to address specific skills deficits identified through that exercise. This requires clinicians to elicit and correctly label events, and to develop an individualized case formulation and corresponding skills training plan. We say ‘at face value’ because research on training in EBT is very limited in general (Frank, Becker‐Haimes, & Kendall, 2020) and virtually nonexistent for the inpatient setting and with nondoctoral level staff, so these assertions need to be confirmed.

Adapting EBTs for inpatient treatment is challenging but crucial to provide adequate care to the thousands of patients who are hospitalized in psychiatric units every year. The prevailing multimodal approach to inpatient treatment applies to psychotherapy as much as it does to other aspects of treatment, and we should embrace flexible and efficient treatment models that do not fit the traditional psychotherapy mold, like strategic treatment add-ons and open, single-session format groups. Combining multiple treatments allows for the balance of generalization and specialization within the restrictions of inpatient care. In practice, these adaptations are already used informally in many hospitals; however, clinicians lack guidance to confirm what works and what does not. We reiterate our call for such research to be conducted.
